# Clinical and Clinico-Pathological Observations of the Erythrocyte Sedimentation Rate in Dogs Affected by Leishmaniosis and Other Inflammatory Diseases

**DOI:** 10.3390/ani14071013

**Published:** 2024-03-27

**Authors:** George Lubas, Saverio Paltrinieri, Roberto Amerigo Papini, Ilaria Lensi, Silvia Lucia Benali, Oscar Cortadellas, Nunzio D’Anna, Alessandra Fondati, Xavier Roura, Eric Zini

**Affiliations:** 1 Clinica Veterinaria Colombo, VetPartners Italia, V.le Colombo 153, 55041 Lido di Camaiore, Italy; lensi.ilaria@gmail.com; 2 Department of Veterinary Medicine and Animal Sciences, University of Milan, Via dell’Università 6, 26900 Lodi, Italy; saverio.paltrinieri@unimi.it; 3 Department of Veterinary Sciences, University of Pisa, c/o Veterinary Teaching Hospital “Mario Modenato”, Via Livornese (Lato Monte), San Piero a Grado, 56122 Pisa, Italy; roberto.amerigo.papini@unipi.it; 4 MYLAV, Laboratorio La Vallonea, Via G. Sirtori, 9, Passirana di Rho, 20017 Milano, Italy; silviabenali@laboratoriolavallonea.it; 5 Hospital Clínico Veterinario, Universidad CEU Cardenal Herrera, 46115 Valencia, Spain; oscar@vetgermanias.com; 6 Clinica Oculistica Veterinaria SeeVet, Via Tuscolana 1709, 00133 Roma, Italy; ndanna@libero.it; 7 Veterinaria Cetego, Via M.C. Cetego 20, 00177 Roma, Italy; alessandrafondati@gmail.com; 8 Hospital Clínic Veterinari, Universitat Autònoma de Barcelona, 08193 Bellaterra, Spain; xavier.roura@uab.es; 9 AniCura Istituto Veterinario Novara, Strada Provinciale 9, 28060 Granozzo con Monticello, Italy; eric.zini@anicura.it; 10 Department of Animal Medicine, Production and Health, University of Padova, Viale dell’Università 16, 35020 Legnaro, Italy; 11 Clinic for Small Animal Internal Medicine, Vetsuisse Faculty, University of Zurich, Winterthurerstrasse 260, 8057 Zurich, Switzerland

**Keywords:** dog, erythrocyte sedimentation rate, leishmaniosis, inflammatory markers, immune response markers

## Abstract

**Simple Summary:**

The erythrocyte sedimentation rate (ESR) has been increasingly used in canine medicine to assess inflammation levels. In this study, the ESR was compared to several inflammatory and immune response markers, typically investigated in dogs with canine leishmaniasis (CanL) and in dogs affected by other inflammatory conditions. Three groups of dogs were included in the study: CanL affected dogs without clinical signs (INFECTED, #25) or with clinical signs (SICK, #43), and dogs affected by acute or acute-on-chronic conditions (OTHER DISEASE, #65). The ESR and several inflammatory (i.e., C reactive protein, fibrinogen, haptoglobin, and ferritin,) or immunological parameters (i.e., total proteins, gamma-globulins, IgG, and IgM) were compared between groups and correlated. The ESR was statistically higher in the SICK group and in the OTHER DISEASE group than in the INFECTED group. ESR values may therefore help to stage the severity of CanL. In addition, as a point-of-care assay, the ESR could be used to screen the health status of dogs with its values being related to the severity of any disease.

**Abstract:**

The erythrocyte sedimentation rate (ESR) has been used in canine medicine in several disorders, above all, to evaluate levels of inflammation. This study evaluated the ESR in canine leishmaniosis (CanL) and other inflammatory conditions. Three groups of dogs were examined: CanL affected dogs without clinical signs (INFECTED group, #25) or with clinical signs (SICK group, #43) and dogs affected by acute or acute-on-chronic conditions (OTHER DISEASE group, #65). The ESR was compared with acute phase proteins or reactants either positive or negative (leukogram, fibrinogen, iron, unsaturated iron binding capacity, ferritin, haptoglobin, and albumin) and immunological markers (gamma-globulins, IgG, and IgM). The ESR was higher in the SICK group than in the INFECTED group (median 39 vs. 11 mm/h; *p* < 0.0001), as well as in the OTHER DISEASE than in the INFECTED groups (median 41 vs. 11 mm/h; *p* < 0.0001). The ESR appeared outside the reference range for all dogs in the SICK and OTHER DISEASE groups and almost with similar values (mm/h; median 39, 95% CI 31–51 vs. 41, 95% CI 12–87; *p* > 0.05). The extent of changes in ESR can help to establish the severity of CanL and other inflammatory disorders. As a point-of-care test, the ESR can be used to screen dogs for unhealthy conditions, and its values correlate with the severity of any disease, including CanL.

## 1. Introduction

The erythrocyte sedimentation rate (ESR) is one of the most common laboratory markers used in human medicine as a generic index of disease, mostly related to the onset and extent of inflammation. The ESR is related to the speed of red blood cell sedimentation in autologous plasma, which is faster in humans in relationship to an increased plasma concentration of certain proteins called “agglomerans”, such as fibrinogen, immunoglobulin M (IgM), and alpha-2-macroglobulin [1,2]. In humans, other factors also influence the speed of aggregation and sedimentation related to the reciprocal effect between the erythrocyte membrane surface and plasma (e.g., hematocrit, albumin, age, sex) [3]. In human medicine, the ESR is commonly increased following acute or chronic tissue damage as well as after many inflammatory conditions such as infections, malignancies, and autoimmune diseases [1,2].

The ESR is now being used again in veterinary medicine after a long period when it had been almost completely abandoned in favor of other inflammatory markers [4,5,6]. In fact, the ESR has been reported in papers dealing with canine osteoarthritis, ehrlichiosis, babesiosis, leishmaniosis, heartworm disease and also for other common health conditions in dogs [7,8,9,10,11,12].

The reference method for ESR measurement is the Westergren method, which constitutes the gold standard as recommended by the International Council for Standardization in Hematology (ICSH). The Westergren method uses a whole blood sample diluted with sodium citrate anticoagulant (4:1), and the value of ESR is determined after one hour in a vertically placed tube [13]. Currently, in human laboratories, modified automated or semiautomated methods are routinely used, using diluted or undiluted samples [14].

In 2020, a modified Westergren ESR assay was validated in dogs (MINI-PET, DIESSE Veterinary, Diagnostica) [6]. The adoption of this semi-automatic system brings numerous advantages, in addition to the reduction in the analysis times, such as a decrease in the costs of the sampling devices, the blood volume necessary for the test and the use of ethylenediaminetetraacetic acid (EDTA) anticoagulant blood that makes it possible to employ the whole blood sample withdrawn for other hematology tests. Two recent published papers in 2022 and 2024 adopted this modified ESR assay [10,11].

The flagellate protozoa *Leishmania infantum* is the main causative agent of canine leishmaniosis (CanL) in Mediterranean countries. Infected dogs are the main reservoir of *L. infantum* in endemic areas, however, the infection can also be transmitted to humans and other mammals, including cats. Although the infection is chronic and systemic, most infected dogs remain without clinical signs. Clinical signs in symptomatic cases vary considerably and serious complications can occur, leading to death if left untreated. Diagnosis is based on clinical signs, abnormal laboratory parameters, serological and molecular techniques, and the cytology of bone marrow/lymphnode aspirates. The treatment consists of leishmanicide and leishmaniostatic drugs and requires great owner compliance as it is time consuming and expensive [15,16].

The goal of this study was to evaluate whether the ESR could be a useful marker to assess the severity of CanL. The aims were thus as follows: (i) to evaluate and compare the ESR values and other immune-inflammatory markers in dogs without or showing clinical signs related to Can-L; (ii) to compare the ESR values and other immune-inflammatory markers between dogs with clinical signs related to Can-L and dogs affected by various acute or acute-on-chronic inflammatory disorders; (iii) and to correlate the ESR values with those of other immune-inflammatory markers in CanL positive dogs and in dogs with various acute or acute-on-chronic inflammatory disorders.

## 2. Materials and Methods

### 2.1. Study Design

A monocentric observational prospective study was performed between October 2021 and September 2023 in a private veterinary clinic. Given that blood and other biological samples were collected for routine diagnostic purposes and solely for the dogs’ benefit, and the owners had signed a consent form that authorized the use of their data and the excess specimens for research purposes, formal approval from the University’s Ethical Committee was not required.

### 2.2. Enrollment of Dogs

Among the patients referred to this clinic, CanL-positive dogs without comorbidities and CanL-negative dogs with an acute or acute-on-chronic inflammatory disorder were enrolled. Dogs with acute-on-chronic inflammatory disorders included those with an acute exacerbation of a chronic disease and those with a new acute disease superimposed on a different and previously existent chronic disease.

Briefly, dogs were examined by two veterinarians (GL and IL) who collected data on signalment through a thorough medical history check and performed a complete physical examination. For each enrolled dog, the following diagnostic workup was also performed. CanL diagnosis was confirmed or ruled out with ELISA, lymph node or bone marrow cytology, or qPCR depending on the clinical presentation of the dogs following the recommendations of the Canine Leishmaniasis Working Group (CLWG) [17,18]. The investigation of diagnostic imaging (abdominal ultrasound and chest radiography) and selected serology or qPCR for other vector borne pathogens (*Ehrlichia canis*, *Anaplasma phagocytophilum*, *A. platys*, *Rickettia* spp., *Babesia* spp., *Bartonella* spp., and *Hepatozoon* spp.) were based on the initial clinical and laboratory findings and performed by the two veterinarians in charge. Concomitant diseases in CanL affected patients were excluded at the time of enrollment and during follow-up, based on the clinico-pathological features investigated.

### 2.3. Number, Signalment, Clinical Classification, and Study Group of Samples Investigated

After completion of all initial clinical and clinicopathological data, three groups of samples were obtained:

SICK group: at the clinical presentation, individual samples were collected from 43 CanL-positive dogs without comorbidities, not treated using any anti-leishmania drug (including antimonials, miltefosine, or allopurinol) in the prior three months, not treated with glucocorticoid in the prior one month, and with clinical and clinico-pathological signs related to leishmaniosis, corresponding to stages C and D of the CLWG classification system [17,18]. Furthermore, all dogs belonging to this group had tested serologically positive for leishmaniosis in their medical history. CanL in SICK dogs was diagnosed with high titer serology (n = 6), lymph node (n = 2) or bone marrow (n = 2) qPCR, and lymph node (n = 2) or bone marrow (n = 2) cytology as well as with a combination of two methods such as medium-high serology associated with lymph node (n = 7) or bone marrow (n = 6) qPCR, and medium-high serology associated with lymph node (n = 9) or bone marrow (n = 7) cytology.

INFECTED group: 57 samples were collected from 25 Can-L positive dogs without comorbidities, with clinical signs and clinico-pathological evidence not directly related to leishmaniosis, corresponding to stage B of the CLWG classification system [17,18]. CanL in INFECTED dogs was diagnosed via serology at the time of enrollment in this group and these were patients already treated with a leishmanicide treatment in the previous months or years. Specifically, 25 individual samples were collected at the first presentation of each dog, while the remaining 32 samples were collected during each of the subsequent checks: one dog had six check-ups, one dog had five check-ups, one dog had four check-ups, two dogs had three check-ups, three dogs had two check-ups, and five dogs had one check-up. The check-ups were not scheduled on a periodic basis but only based on the owner’s availability, about 2–3 months apart from each other.

OTHER DISEASE group: each sample was collected from 65 Can-L negative dogs, without any clinical signs related to an active form of Can-L, with negative serology for leishmaniosis, and with acute or acute-on-chronic inflammatory disorders sampled at first clinical presentation.

Table 1 reports the signalment data for all the enrolled dogs. Table 2 reports the main clinical features of the 43 SICK Can-L positive dogs along with the corresponding CLWG stages at the time of enrollment. Table 3 reports the list of diseases or the main clinical signs of the 65 dogs belonging to the OTHER DISEASE group.

### 2.4. Laboratory Assays

Blood samples were collected from the jugular vein and divided in three types of tubes: K3-EDTA, for CBC and ESR assays; sodium citrate at 3.8% for fibrinogen; and plain without any additive or gel for serum biochemistry analytes.

The ESR was determined on 1 mL K3-EDTA vials (APTACA S.p.A., Canelli, Italy) using MINI-PET (DIESSE Veterinary, Diagnostica Senese S.p.A., Siena, Italy). MINI-PET works without blood consumption, thus, if during the use of this device an ‘error’ was reported (less than 1% of readings), the reading was then repeated once again immediately after gently mixing the vial using inversion at least 10 times. The ESR samples were assayed within one hour from the blood collection, after the blood cell count had been carried out.

In order to fulfil the aims of this study, only the following parameters were considered: Hematocrit (Hct) and total leukocyte count (WBC) taken from the CBC (Idexx ProCyte^®^ Dx laser cell counter, Idexx Laboratories, Westbrook, ME, USA); neutrophil band count (Bands) evaluated in the manual differential leukocyte count performed by an experienced clinical-pathologist [GL], from stained blood smears (May–Grundwald Giemsa stain, MGG Quick Stain, Bio-Optika, Milan, Italy); C-reactive protein (CRP), Iron, Unsaturated Iron Binding Capacity (UIBC), Ferritin, Haptoglobin (HPT), Total proteins, Albumin, Immunoglobulin G (IgG), and Immunoglobulin M (IgM) from the serum biochemistry (AU 5800, Beckman Coulter, Inc., Brea, CA, USA, with dedicated kit reagents). Albumin/Globulin (A/G) ratios were also calculated. The Gamma-globulin (SPE gamma) percentage was obtained from serum electrophoresis (Capillarys Tera, Sebia, Evry Cedex, France) and Fibrinogen from the coagulation profile was also recorded (BCS XP, Siemens Healthcare Diagnostics, Marburg, Germany, with dedicated kit reagents).

The serology for *Leishmania* was carried out with the Leiscan^®^ *Leishmania* ELISA test (Hipra, Ecuphar Italia srl, Milan, Italy) [19]. The assay was carried out in the serum following the manufacturer’s protocol. The results were calculated and classified as follows: (Razon, Rz, of the sample = sample Optical Density/ control low positive sample Optical Density) < 0.7 negative; 0.7–1.5 suspected; 1.5–3 low positive; 3–6 medium positive; >6 high positive. The test has shown a sensitivity of 92.5–98% and a specificity of 100% in comparison studies [19,20] and has been successfully used for a previous serological survey in dogs in Spain [21]. The qPCR for *Leishmania* was carried out from lymph node or bone marrow biopsy samples according to the method described by Castelli et al., 2021 [22]. The detection limit was set at 100 copies of kinetoplast.

### 2.5. Statistical Analysis

All the laboratory parameters were statistically evaluated as follows: (I) measurement values in samples from the groups of INFECTED, SICK and OTHER DISEASE were compared each other; and (II) ESR results were correlated with all the other investigated parameters in the three study groups. Unfortunately, the measurements of some laboratory parameters were lacking, as reported in detail in Table 4 and Table 5.

Differences in signalment between the INFECTED; SICK and OTHER DISEASE groups were statistically investigated using the Chi-squared test with respect to breed, sex, and reproductive status, and using the Mann–Whitney test with respect to age.

All the above blood parameters in dogs in the SICK, INFECTED, and OTHER DISEASE groups were assayed for normal distribution with the D’Agostino–Pearson test. All the values determined for each analyte were considered as non-parametric data, and were reported as median, lowest, and highest value, and with a 95% confidence interval for the median. For each blood parameter in the SICK, INFECTED, and OTHER DISEASE groups, the percentage of values inside or outside the reference interval was also calculated.

The Mann–Whitney test (independent samples, data not normally distributed, as assessed with the D’Agostino–Pearson test) was used to compare blood parameter values for samples from the three groups, i.e., INFECTED, SICK and OTHER DISEASE.

The Spearman rank correlation test (interpretation of rho: 0.1–0.3 weak, 0.4–0.6 moderate, and 0.7–0.9 strong) was used to correlate the ESR values to those of all the blood parameters investigated (Hct, WBC, Bands, CRP, Iron, UIBC, Ferritin, HPT, Total proteins, Albumin, A/G, SPE gamma, IgG, IgM, and Fibrinogen) in the three groups of dogs (INFECTED, SICK, and OTHER DISEASE). This test can provide a positive or negative correlation, i.e., the increase in ESR value is related to the increase or decrease, respectively, in the value of the blood parameter examined.

All the statistical analyses were carried out using MedCalc (v. 15.11, Ostend, Belgium), and the *p* value was set at 0.05.

## 3. Results

The results of the ESR test carried out using MINI-PET were simple and relatively fast, using the same vial of blood in K3-EDTA just after processing the sample through the blood cell counter.

### 3.1. Differences in Signalment Data in the Three Group of Dogs

The signalment data in the three groups of dogs were different from each other. There were more mixed breeds in the INFECTED group (14/25, 56.0%) and in the SICK group (23/43, 53.5%) than the OTHER DISEASE group (28/65, 43.1%), but this difference was not statistically significant (Chi-squared test, *p* ≥ 0.05). There were more males and fewer females in the INFECTED group (males 15/25, 60.0%; females 10/25, 40.0%) and in the SICK group (males 33/43, 76.7%; females 10/43, 23.3%) in comparison to the OTHER DISEASE group (males 29/65, 44.6%; females 36/65, 55.4%) and the difference was statistically significant (Chi-squared test, *p* = 0.004). The median age was significantly higher in the OTHER DISEASE group (9 years) than in the SICK (5 years) and INFECTED (6 years) groups and the differences were statistically different (Mann–Whitney test, for both, respectively *p* = 0.0001).

### 3.2. Comparison of Measurements in INFECTED, SICK and OTHER DISEASE Groups

Table 4 shows values of the laboratory parameters investigated in the samples of the INFECTED, SICK, and OTHER DISEASE groups. We chose to use a statistical test for data not normally distributed as the results can be reported uniformly, i.e., median, lowest, and highest value, and confidence interval. Indeed, the only parameters that showed data normally distributed in the three groups were Hct, Albumin, and A/G ratio.

**Table 4:** ESR values and other laboratory parameters in blood samples from dogs in the INFECTED, SICK and OTHER DISEASE groups and the statistical comparative evaluation.

Parameter and Units	Reference Interval	INFECTED Group		SICK Group		OTHER DISEASE Group	
		N	Median (95% CI) Min–Max	N	Median (95% CI) Min–Max	N	Median (95% CI) Min–Max
ESR (mm/h)	<10	57	11 (10–11) a *** 2–15	43	39 (31–51) b ^ns^ 11–77	65	41 (31–44.0) c *** 12–87
Hematocrit %	37.3–61.7	57	45.6 (44.7–46.7) a *** 33.2–57.5	43	34.6 (31.3–37.6) b ^ns^ 16.5–49.7	65	32.9 (29.1–35.4) c *** 9.1–56.6
WBC K/µL	5.05–16.76	57	9.29 (8.66–9.84) a ^ns^ 3.51–19.7	43	8.80 (7.14–10.5) b ** 3.56–38.5	65	12.2 (10.8–15.4) c *** 1.4–177.7
Bands K/µL	0.0–0.3	57	0.00 (0.00–0.00) a * 0.00–0.21	43	0.00 (0.00–0.00) b ** 0.00–1.79	65	0.07 (0.00–0.14) c *** 0.00–4.84
CRP mg/L	0–0.15	57	0.80 (0.56–1.19) a *** 0.04–13.6	43	7.6 (2.2–11.9) b ^ns^ 0.0–33.6	65	7.6 (4.0–13.5) c *** 0.1–48.0
Fibrinogen mg/dL	104–342	56	199 (179–214) a *** 103–425	43	358 (302–429) b ^ns^ 163–865	65	345 (309–487) c *** 30–794
Iron µg/dL	70–270	56	106 (99–121) a *** 59–362	43	80 (69–90) b *** 13–260	64	123 (95–148) c ^ns^ 6–364
UIBC µg/dL	156–383	55	244 (223–267) a * 121–395	42	214 (191–231) b ^ns^ 18–379	65	220 (147–253) c * 1.0–429
Ferritin ng/mL	95–287	56	324 (246–367) a *** 95–792	42	558 (402–662) b ^ns^ 136–3235	63	474 (365–588) c *** 87–5289
HPT mg/dL	18–117	37	93 (55–107) a *** 17–398	36	187 (122–230) b ** 36–599	59	284 (219–297) c *** 50–917
Total proteins g/dL	5.5–7.6	57	6.71 (6.53–6.99) a *** 5.32–8.42	43	7.59 (7.26–8.71) b *** 4.5–13.3	65	6.2 (6.0–6.45) c ** 3.7–10.7
Albumin g/dL	2.4–3.8	57	3.01 (2.91–3.11) a *** 2.23–3.59	43	2.24 (1.97–2.63) b * 1.20–3.69	65	2.63 (2.54–2.79) c *** 1.20–3.85
A/G NA	0.6–1.3	57	0.80 (0.77–0.90) a *** 0.44–1.33	43	0.41 (0.30–0.50) b *** 0.15–1.03	65	0.74 (0.65–0.82) c ** 0.24–1.16
SPE gamma %	5–15	56	12.6 (11.5–14.3) a *** 8.9–39.9	43	33.2 (20.1–41.9) b *** 10.3–59.9	65	13.0 (10.2–13.6) c ^ns^ 5.1–58.6
IgG mg/dL	307–787	52	658 (546–797) a *** 329–2238	40	1399 (900–2128) b *** 468–4608	59	464 (420–556) c *** 126–3030
IgM mg/dL	64–176	49	143 (123–176) a ^ns^ 69–318	39	157 (135–204) b *** 54–616	57	102 (89.6–126.4) c ** 23–648

Legend: ESR, erythrocyte sedimentation rate; WBC, total leukocyte count; CRP, C-reactive protein; UIBC, unsaturated iron binding capacity; HPT, haptoglobin; A/G, albumin–globulin ratio; SPE gamma, gamma globulin in serum protein electrophoresis; IgG, immunoglobulin G; IgM, immunoglobulin M; NA, not applicable; N, number of samples tested, and comparison statistically evaluated; CI, confidence interval; Min-Max, minimum and maximum values. Statistics: a, group comparison INFECTED vs. SICK; b, group comparison SICK vs. OTHER DISEASE; c, group comparison INFECTED vs. OTHER DISEASE; ns, not significant; * *p* < 0.05 and 0.01; ** *p* < 0.01 and 0.001; *** *p* < 0.001.

When the SICK group was compared with the INFECTED group, ESR, Bands, CRP, Fibrinogen, UIBC, Ferritin, HPT, Total proteins, SPE gamma, and IgG were significantly increased while Hct, Iron, Albumin, and A/G were significantly decreased (mostly at *p* < 0.0001, except for Bands and UIBC with *p* between <0.05 and 0.01).

When the SICK group was compared with the OTHER DISEASE group, Total proteins, SPE gamma, IgG, and IgM were significantly higher, while WBC, Bands, Iron, HPT, Albumin, and A/G were significantly lower (mostly at *p* < 0.0001, except for WBC, Bands, and HPT with *p* between <0.01 and 0.001, and Albumin with *p* between <0.05 and 0.01).

When the OTHER DISEASE group was compared with the INFECTED group, ESR, WBC, Bands, CRP, Fibrinogen, Ferritin, IgG, and IgM were significantly higher, while Hct, UIBC, HPT, Total proteins, Albumin, and A/G were significantly lower (mostly at *p* < 0.0001, except for Total proteins, A/G, and IgM with *p* between <0.01 and 0.001, and UIBC with *p* between <0.05 and 0.01).

**Figure 1:**
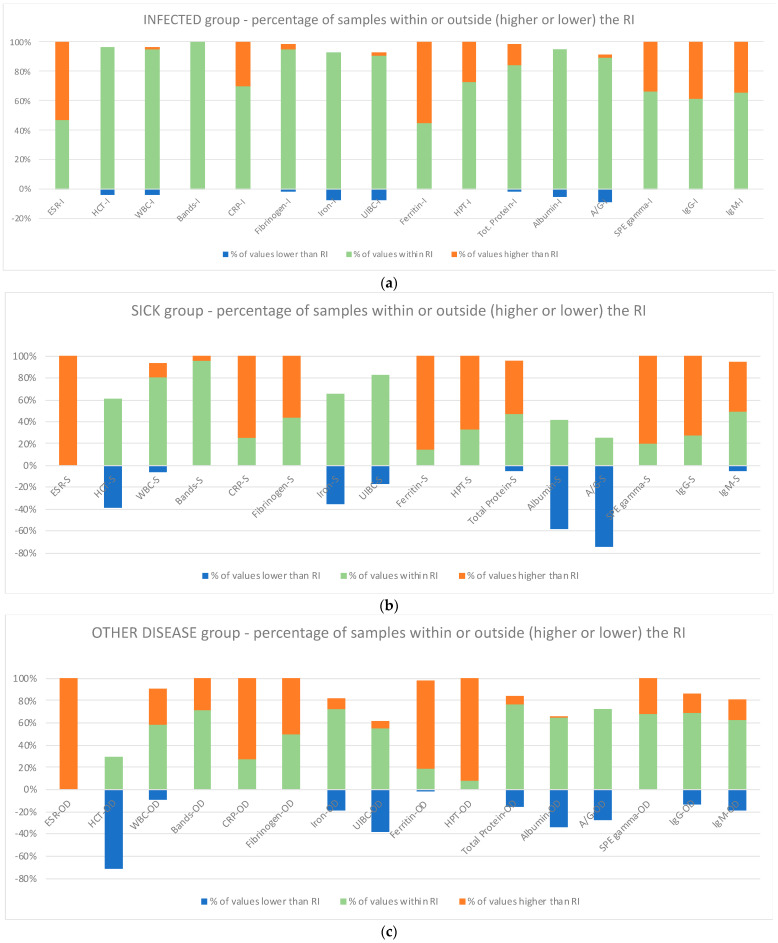
The analytes investigated (ESR, HCT, WBC, Bands, CRP, Fibrinogen, Iron, UIBC, Ferritin, Haptoglobin, Total proteins, Albumin, A/G ratio, SPE gamma, IgG, and IgM) in the INFECTED (**a**), SICK (**b**), and OTHER DISEASE (**c**) groups evidenced with different colors for values lower and higher than RI or within RI and plotted. Legend: the letter after each blood parameter identifies the various group: I, Infected; S, Sick; OD, Other Disease; ESR, erythrocyte sedimentation rate; WBC, total leukocyte count; CRP, C-reactive protein; UIBC, unsaturated iron binding capacity; A/G, albumin–globulin ratio; SPE gamma, gamma globulin in serum protein electrophoresis; IgG, immunoglobulin G; IgM, immunoglobulin M.

The percentage of values within or outside the reference intervals for all measured parameters is reported in Figure 1.

In the INFECTED group, values outside the reference interval were below 50% for all parameters, except for ESR and Ferritin (with higher values).

In the SICK group, the highest percentage of values falling outside the reference interval (more than 50% and in decreasing order) was found for ESR, Ferritin, SPE gamma, CRP, IgG, HPT, Fibrinogen, Total proteins, and IgM (with higher values) and A/G, Hct, and Albumin (with lower values).

In the OTHER DISEASE group, the highest percentage of values falling outside the reference interval (more than 50% and in decreasing order) was found for ESR, HPT, Ferritin, CRP, and Fibrinogen with higher values except for Hct for lower values.

### 3.3. Correlation between ESR Values and Other Laboratory Parameters

Table 5 shows the statistical analysis of the ESR values in comparison with the values of all parameters investigated in the dogs of the INFECTED, SICK and OTHER DISEASE groups, using the Spearman rank correlation test. A significant negative correlation was found between the ESR and Hct in all groups. In the SICK group, the ESR level also correlated positively with Fibrinogen and Bands, and negatively with Iron, Albumin, and A/G. In the OTHER DISEASE group, the ESR level correlated positively with Fibrinogen, Bands, and CRP, and negatively with UIBC and A/G.

**Table 5:** Relationship between ESR and other laboratory parameters in blood samples of dogs in the group of INFECTED (57 samples), SICK (43 samples), and OTHER DISEASE (65 samples), as determined through the Spearman rank correlation test.

ESR vs. Parameters	INFECTED		SICK		OTHER DISEASE	
	rho	*p*	rho	*p*	rho	*p*
Hematocrit	−0.608	***	−0.741	***	−0.384	***
WBC	0.010	ns	0.212	ns	−0.179	ns
Bands	0.096	ns	0.323	*	0.388	***
CRP	0.247	ns	0.210	ns	0.374	**
Fibrinogen	0.175	ns	0.524	***	0.449	***
Iron	0.046	ns	−0.465	**	0.109	ns
UIBC	−0.093	ns	−0.200	ns	−0.278	*
Ferritin	−0.109	ns	0.228	ns	0.163	ns
Haptoglobin	0.070	ns	−0.069	ns	0.213	ns
Total proteins	−0.030	ns	−0.019	ns	0.172	ns
Albumin	−0.113	ns	−0.530	***	−0.234	ns
A/G	−0.005	ns	−0.359	*	−0.363	**
SPE gamma	0.225	ns	0.297	ns	0.180	ns
IgG	0.019	ns	0.157	ns	0.125	ns
IgM	0.188	ns	0.052	ns	0.050	ns

Legend: ESR, erythrocyte sedimentation rate; WBC, total leukocyte count; CRP, C-reactive protein; UIBC, unsaturated iron binding capacity; A/G, albumin–globulin ratio; SPE gamma, gamma globulin in serum protein electrophoresis; IgG, immunoglobulin G; IgM, immunoglobulin M; ns, not significant; negative values indicate a negative correlation (values of this parameter were increased in parallel to the decrease in ESR values); positive values indicate a positive correlation (values of this parameter were increased in parallel to the increase in ESR); * *p* < 0.05 and 0.01; ** *p* < 0.01 and 0.001; *** *p* < 0.001.

## 4. Discussion

In this study, the ESR test was successfully used in dogs affected by leishmaniosis at the time of diagnosis, as well as for monitoring those dogs affected by a severe form of the disease during the leishmanicide treatment and during the monitoring of asymptomatic dogs. ESR is a modified assay and point-of-care test in dogs and can be carried out with the same vial used for CBC (1 mL of blood with K3-EDTA added) within six hours from sampling, when the sample is stored at room temperature, or up to 24 h when stored in a refrigerator [23]. The MINI-PET device only generated an error message in less than 1% of cases, however, this was easily resolved through inverting the vial for another thorough mixing and measuring it again [23]. The value of 10 mm/h which was used as the upper limit of the reference interval in this study, as recommended by Militello et al. [6], seems to be more appropriate to classify healthy dogs than the lower reference limit (8 mm/h) recently proposed by Gori et al. [24].

To assess the potential utility of the ESR, SPE gamma, IgG, IgM and positive or negative acute phase proteins were evaluated simultaneously. These parameters are widely used to evaluate the severity of CanL [17,25,26,27,28,29,30,31,32,33].

We found that dogs in the INFECTED group had only moderate signs of residual inflammation or immune response, with a complete absence of detectable clinical signs related to CanL, since median hematological and biochemical values were outside the reference intervals only in a small number of dogs. The only exceptions were represented by ESR and ferritin, for which a high proportion of patients still had higher values compared with the reference interval (respectively 55% and 53%). It is well known that ferritin may be a very informative marker of CanL disease [25,26], however, several other factors such as anemia or chronic or intercurrent inflammation may induce hyperferritinemia.

On the other hand, for almost all the hematological and biochemical analytes, both the SICK and the OTHER DISEASE groups showed preliminary changes, both in terms of median and min-max values and in the frequency of values outside the reference intervals. The results from these two groups did not differ significantly from each other, although changes in biomarkers, indicative of an immune-mediated origin of inflammation (i.e., gamma globulins and IgG), were more evident in the SICK group than in the OTHER DISEASE group. Despite this last difference, the analysis of these preliminary clinico-pathological results of the SICK and OTHER DISEASES groups confirms that the SICK dogs presented the typical changes consistent with clinically evident CanL. In addition, results from the OTHER DISEASE group, despite its heterogeneous composition in terms of type and severity of disease and the significantly lower median age in comparison to the SICK group, preliminarily confirm that the OTHER DISEASE group was adequate to compare the ESR values recorded in dogs with CanL as they were a group of dogs with a similar severity of inflammation and anemia.

Based on the group composition and routine clinical pathology findings, it is therefore not surprising that a low percentage of patients with increased ESR were detected, compared with the reference intervals, in the INFECTED group. This is because the individual dogs in this group showed slight changes compared to the hematological and biochemical reference intervals, probably due to residual minor inflammatory changes.

By contrast, the ESR was significantly higher than the reference intervals in nearly all the dogs from the SICK and OTHER DISEASE groups compared to the INFECTED group. This result is also not surprising, since both groups showed clear hematological and biochemical changes regarding the parameters that have been reported to induce higher ESR. For example, a mild-to-severe decrease in Hct was detected in both groups, and the ESR was strongly negatively correlated with Hct in all groups, albeit with a different level of statistical significance. It is also well known that the low Hct is highly correlated with a higher ESR [34,35].

In human medicine, in fact, a correction of the ESR based on the value of the Hct has been proposed [36,37]. In veterinary medicine, this correction has not been studied to date although it has already been proposed [6]. However, it might be recommended in the future in order to eliminate the possible effect of anemia in the analysis of data from dogs with and without inflammation.

Despite the possible effect of a lower Hct, inflammation seems to be the main trigger for the higher ESR in both SICK and OTHER DISEASE dogs. In fact, the ESR in SICK dogs correlated negatively to Albumin, which is a negative APP, and positively correlated to Fibrinogen, a positive APP [29,30]. On the other hand, in dogs with OTHER DISEASES, ESR correlated with markers of acute inflammation such as Bands, CRP, Fibrinogen, and HPT.

The ESR was outside the RIs in all dogs in these two groups, suggesting that the ESR increase is not specific to a given disease but is non-specifically related to an unhealthy status. However, the SICK group had the highest ESR values, which were significantly higher than those recorded in the OTHER DISEASE group. Despite the similar inflammatory pattern, this suggests that the major increase in ESR in the SICK group may be due to clinically evident leishmaniosis. However, the design of this study prevents this hypothesis from being confirmed, or from hypothesizing a possible pathogenic mechanism.

Further studies, possibly with a control group made up of inflammatory and immune-mediated diseases, are needed to understand whether the triggering mechanism for higher ESR in dogs with leishmaniosis is the most intense activation of the immune system, as suggested via clinico-pathological tests, or whether a different mechanism is involved. In addition, further research could investigate whether there is a possible cut-off for differentiating between dogs with inflammation associated with leishmaniosis and dogs with inflammation due to other diseases.

This study has some limitations typical of a clinical study collecting cases in the field.

First, serology with ELISA was not carried out at the enrollment of all dogs included in the SICK group as they were already tested positive serologically in the history (with different methods, i.e., immunofluorescence antibody test, and techniques) and so other investigations such as cytology or qPCR were preferably used.

Second, the clinical signs of dogs belonging to the SICK group were different in the CLWG stage and type of the main clinical signs. Of course, we think this variability could influence the clinico-pathological data as well. A group of dogs with similar clinical signs would be more accurate to compare. In addition, we did not use any clinical rating scale for clinical signs as reported by Da Silva et al. or by Miro et al. [38,39].

Third, the clinical signs of dogs belonging to the OTHER DISEASE group were different having only the main problem as an acute or acute-on-chronic condition. A group of dogs with the same disease or with the same grading of inflammation would be more accurate to compare. In addition, several dogs mainly affected by immune-mediated disorders were previously or currently treated with glucocorticoid or immune-suppressive drugs that could affect the results of several blood parameters investigated in this study.

## 5. Conclusions

The ESR results outside the RI in the dogs investigated in this study suggest an unhealthy condition due to a disease. The comparison between SICK dogs and dogs with OTHER DISEASES highlighted different inflammation patterns. In the SICK dogs, this was due to a combination of immune reaction and inflammation, while in the OTHER DISEASES dogs it was only due to both acute and acute-on-chronic inflammation.

Since ESR is a point-of-care assay, it could be used to screen dogs for the unhealthy conditions, followed by additional tests for the diagnosis of disease. The magnitude of ESR would likely reflect the degree of inflammation or the severity of the disease. In addition, the values of ESR could be used to monitor the improvement or worsening of the inflammatory process.

It is suggested that ESR can potentially be one of the most valuable laboratory markers in the assessment of CanL on presentation alongside its use in the identification of an inflammation status in the patients. Nonetheless, although these preliminary data are promising, they deserve further investigations and other prospective studies are needed to better elucidate the value of ESR to monitor CanL treatment.

## Figures and Tables

**Table 1:** Signalment data for enrolled dogs: 43 SICK Can-L positive dogs with clinical and clinico-pathological signs related to leishmaniosis; 25 INFECTED Can-L positive dogs without clinical and clinico-pathological signs related to leishmaniosis; 65 OTHER DISEASE Can-L negative dogs with acute or acute-on-chronic inflammatory disease other than leishmaniosis.

Breed	N	Age	Sex and Reproductive	N
SICK Can-L positive dogs (N = 43)			status	
Mixed	23	Median 5 years	Males	32
English setter	5	Range 2–14 years	Males castrated	1
French Bouledogue	3		Females	3
Boxer, Siberian husky (two each)	4		Females spayed	7
American Staffordshire, Brittany spaniel, Corso, Dobermann, Galgo, Rough collie, Yorkshire terrier, Whippet (1 each)	8			
INFECTED Can-L positive dogs (N = 25)				
Mixed	14	Median 6 years	Males	13
English setter, French Bouledogue (two each)	4	Range 2–10 years	Males castrated	2
Bull terrier, Boxer, Chihuahua, Corso, Italian greyhound, Rottweiler, Yorkshire terrier (1 each)	7		Females Females spayed	4 6
OTHER DISEASE Can-L negative dogs (N = 65)				
Mixed	28	Median 8.9 years	Males	20
Cocker spaniel	6	Range 2–17 years	Males castrated	9
Labrador retriever	4		Females	4
Bernese, Boxer, Dachshund, English setter, German shepherd, Jack Russell terrier, Rottweiler (two each)	14		Females spayed	32
Alaskan malamute, American Staffordshire, Beagle, Bolognese, Cavalier King Charles spaniel, Czechoslovakian Wolfdog, Golden retriever, Irish setter, Maltese, Pomeranian, Poodle, Schnauzer, Whippet (one each)	13			

N—number of dogs.

**Table 2:** Main clinical features and CLWG clinical stage of 43 dogs with leishmaniosis (SICK) at the time of enrollment.

Main Clinical Problem/s or Sign/s	N	CLWG Stage
Skin disease and lymphadenopathy	6	
Weight loss and lymphadenopathy	4	
Chronic renal failure, polyarthritis and lymphadenopathy, weight loss and epistaxis (three each)	9	D = 28
Skin disease and enteropathy, skin disease and weight loss, skin disease (two each)	6	
Weight loss and enteropathy, epistaxis, uveitis and lymphadenopathy, (one each)	3	
Weight loss	7	
Weight loss and enteropathy	3	C = 15
Enteropathy, enteropathy and lymphadenopathy, lymphadenopathy, polyarthritis, weight loss and skin disease, (one each)	5	

N—number of dogs.

**Table 3:** List of diseases of the 65 dogs with acute or acute-on-chronic inflammatory diseases at the time of enrollment.

Disease/s or Main Clinical Problem/s	N
Acute inflammation *	15
Chronic enteropathies with acute/subacute relapse	12
Bone marrow dysplasia involving RBC or PLT, immune mediated thrombocytopenia, (five each)	10
Head neoplasia, immune mediated hemolytic anemia, porto-systemic shunt (three each)	9
Arthropathy, histiocytic sarcoma, hyperadrenocorticism with acute inflammation, immune mediated polyarthritis, lymphoma, myeloid leukemia, non-regenerative anemia with subacute inflammation (two each)	14
Cholecystitis, Evan’s syndrome, hypothyroidism, liver disease, myeloma (one each)	5

N—number of dogs; note: * skin abscess N 4, bite injuries N 3, severe stomatitis N 2, severe pyoderma N 2, pyometra N 2, bronchopneumonia N 1, pyothorax N 1.

## Data Availability

Data about this research are available on request to G.L. and are covered by privacy.
